# Association of blood pressure after peritoneal dialysis initiation with the decline rate of residual kidney function in newly-initiated peritoneal dialysis patients

**DOI:** 10.1371/journal.pone.0254169

**Published:** 2021-07-08

**Authors:** Yusuke Kuroki, Kei Hori, Kazuhiko Tsuruya, Dai Matsuo, Koji Mitsuiki, Hideki Hirakata, Toshiaki Nakano, Takanari Kitazono

**Affiliations:** 1 Nephrology & Dialysis Center, Japanese Red Cross Fukuoka Hospital, Fukuoka, Japan; 2 Division of Nephrology, Munakata Medical Association Hospital, Fukuoka, Japan; 3 Department of Nephrology, Nara Medical University, Nara, Japan; 4 Department of Medicine and Clinical Science, Graduate School of Medical Sciences, Kyushu University, Fukuoka, Japan; Kawasaki Ika Daigaku, JAPAN

## Abstract

**Background:**

Lower blood pressure (BP) levels are linked to a slower decline of kidney function in patients with chronic kidney disease (CKD) without kidney replacement therapy. However, there are limited data on this relation in peritoneal dialysis (PD) patients. Here we evaluated the association of BP levels with the decline of residual kidney function (RKF) in a retrospective cohort study.

**Methods:**

We enrolled 228 patients whose PD was initiated between 1998 and 2014. RKF was measured as the average of creatinine and urea clearance in 24-hr urine collections. We calculated the annual decline rate of RKF by determining the regression line for individual patients. RKF is thought to decline exponentially, and thus we also calculated the annual decline rate of logarithmic scale of RKF (log RKF). We categorized the patients’ BP levels at 3 months after PD initiation (BP_3M_) into four groups (Optimal, Normal & High normal, Grade 1 hypertension, Grade 2 & 3 hypertension) according to the 2018 European Society of Cardiology and European Society of Hypertension Guidelines for the management of arterial hypertension.

**Results:**

The unadjusted, age- and sex-adjusted, and multivariable-adjusted decline rate of RKF and log RKF decreased significantly with higher BP_3M_ levels (P for trend <0.01). Compared to those of the Optimal group, the multivariable-adjusted odds ratios (95% confidence interval) for the faster side of the median decline rate of RKF and log RKF were 4.04 (1.24–13.2) and 5.50 (1.58–19.2) in the Grade 2 and 3 hypertension group, respectively (p<0.05).

**Conclusions:**

Higher BP levels after PD initiation are associated with a faster decline in RKF among PD patients.

## Introduction

Regardless of the dialysis modality, the preservation of residual kidney function (RKF) is an independent predictor of survival in patients with chronic kidney disease (CKD) G5D [1–6]. Even in peritoneal dialysis (PD) patients, who have a low glomerular filtration ratio, the preservation of RKF is associated with the following beneficial effects: increased sodium removal, improved volume status [7], reduced left ventricular hypertrophy [8], higher clearance of β-2 microglobulin [9], and improved nutritional status [10]. Even a slight decline in RKF impacts a PD patient’s survival, and it is thus important to be able to predict a decline in RKF.

Several factors that influence the decline of RKF during PD therapy have been revealed. Increased proteinuria [11] and hyperuricemia [12] were significantly associated with a faster decline in RKF, while angiotensin converting enzyme (ACE) inhibitors and angiotensin II receptor blockers (ARBs) slowed the decline of RKF [13]. Therapeutic interventions targeting these factors might help combat the decline of RKF.

Hypertension is a strong risk factor for the progression of CKD [14], and thus treatment of hypertension is the basis of the control of CKD progression. It was also reported that lower blood pressure (BP) levels were associated with a slower decline in kidney function in patients with CKD without kidney replacement therapy [15–17]. Insufficient BP control is an important risk factor for CKD G5D [18]. However, there are limited data on this association in PD patients. We conducted the present retrospective cohort study to evaluate the association between BP levels and the decline rate of RKF.

## Materials and methods

### Study population

The subjects were 178 patients who began PD therapy at Munakata Medical Association Hospital and 89 patients at Kyushu University Hospital between August 1998 and March 2014 (total 267 patients), whose data was obtained from the records in March 2016. None of the patients had undergone kidney transplantation or had switched from hemodialysis to PD. Four patients who were followed-up for <6 months were excluded. Thirty-five patients were excluded because of inappropriate data or because RKF measurement was obtained less than twice. As a result, 228 patients were included in this study. The study protocol was approved by the Local Ethics Committee of Kyushu University Hospital (No. 21–16) and was registered at the University Hospital Medical Information Network (UMIN000018902). For those who could be contacted, written consent was informed. However, for those who could not be contacted because of the end of hospital visit or death, written consent was not informed. For those who could not, we applied opt-out method on this retrospective study. The ethics committee specifically approved our opt-out method of consent for patients who were not reachable, which contents are specified in the research proposal. And also, the analysis used anonymous clinical data.

### Data collection

The following demographic, clinical, and biochemical parameters were assessed just before the start of each patient’s dialysis therapy: age, sex, presence/absence of diabetes mellitus, body mass index (BMI), previous history of cardiovascular disease (CVD), blood hemoglobin, serum albumin, and serum creatinine. The BMI was calculated as the weight in kilograms divided by the square of height in meters. The estimated glomerular filtration rate (eGFR) was calculated using the equation developed by the Japanese Society of Nephrology: eGFR = 194 × sCr^−1.094^ × age^−0.287^ (for females × 0.739) [19]. The dialysate-to-plasma creatinine ratio at 4 hr (D/P creat) was obtained from 1 month after PD initiation. Information about the use of medications and the following dialysis parameters were collected from 3 months after PD initiation: the daily dialysate volume, the use of a high-glucose concentration of PD fluid, and the use of icodextrin. A high glucose concentration of PD fluid was defined as ≥2.5%. The incidence of peritonitis is expressed as the frequency per year during the patient’s PD therapy.

### Blood pressure measurement and classification

BP was assessed with the patient in the sitting position during routine examinations at our outpatient department at the time point of 3 months after PD initiation (BP_3M_). BP measurements were obtained using a standard mercury sphygmomanometer or an automated device. Subjects were categorized into the following four groups according to the 2018 European Society of Cardiology and European Society of Hypertension Guidelines for the management of arterial hypertension [20]: Optimal (O; <120/80 mmHg), Normal & High normal (N; 120–139/80–89 mmHg), Grade 1 hypertension (G1; 140–159/90–99 mmHg), and Grade 2 & 3 hypertension (G2–3; ≥160/≥100 mmHg). Patients whose systolic and diastolic BP indicated different categories were categorized into the higher category.

### RKF, log RKF, and the decline rates of RKF and log RKF

RKF was measured as the mean urea and creatinine clearance values of a 24-hr urine collection after correcting for the patient’s body surface area using the Du Bois formula [21, 22]. Collections of <100 ml were defined as anephritic [11] so that when a urine collection was <100 ml, subsequent collections of <100 ml were excluded from the analysis. RKF was prospectively measured within 3 months of PD initiation, and at 4− 6-month intervals thereafter. We calculated the annual rate of the decline of RKF from serial RKF measurements by creating regression lines for individual patients. The slope of the line through two data points was used in patients with less than three measurements. RKF is considered to decline exponentially, and thus the rate of logarithmic RKF (log RKF) decline is more linearly correlated with time [12,23,24]. Therefore, the decline rate of log RKF was also calculated in the same manner as the decline rate of RKF.

### Statistical analyses

The statistical analyses were performed using the JMP program, ver. 11.0 (SAS, Cary, NC). Results are expressed as the numbers of patients (percentages) or frequencies, or as the median and interquartile range (IQR) (first to third quartiles) for continuous variables. The linear trends in the means and the frequencies of risk factors across BP categories were tested using a linear regression analysis and Cochran-Armitage trend test, respectively. The multivariate-adjusted means of the decline rates of both RKF and log RKF according to BP levels were calculated with an analysis of covariance (ANCOVA) and compared with Dunnett’s t-test. The odds ratio (OR) with the 95% confidence interval (CI) for a faster decline in RKF (i.e., an RKF decline rate faster than the median RKF decline rate) was analyzed by univariate and/or multivariable-adjusted logistic regression. The OR for a faster log RKF decline rate was analyzed in the same manner as the RKF decline rate. The variance inflation factor (VIF) of all parameters was checked in all multivariate analyses in order to avoid multicollinearity. Values of p<0.05 were considered significant in all analyses.

## Results

The overall median (IQR) decline rate of RKF and that of log RKF were −1.09 (−1.93– −0.59) ml/min/1.73m^2^/year and −0.54 (−1.08– −0.23) log(ml/min/1.73m^2^/year), respectively. The median age of patients was 62 (52–72) years. There were 147 males (64.5%) and 78 diabetics (34%). The baseline subject characteristics according to BP categories are shown in Table 1. There were 27, 57, 75 and 69 subjects in the O, N, G1 and G2–3 groups, respectively. The mean eGFR, hemoglobin, frequency of icodextrin use, diuretics use and peritonitis rate were significantly decreased with higher BP_3M_ levels. The uses of RAS inhibitors and diuretics were not different between 1 month and 3 months after the initiation of PD.

**Table 1:** Baseline characteristics of the patients by the graded BP groups.

Variables	O (n = 27)	N (n = 57)	G1 (n = 75)	G2–3 (n = 69)	P for trend
Age (years)	60 (45–75)	66 (58–72)	62 (53–73)	60 (51–70)	0.22
Female	33	32	28	48	0.10
Diabetes mellitus	44	26	39	32	0.71
Previous history of CVD	15	19	20	10	0.35
eGFR (ml/min/1.73m^2^)	5.4 (5.0–6.6)	4.9 (3.9–6.1)	4.8 (4.0–5.9)	4.7(3.7–5.6)	0.012
Hemoglobin (g/dl)	9.5 (8.0–10.4)	9.0 (8.2–9.8)	9.1 (7.8–10.0)	8.4 (7.4–9.4)	0.01
Albumin (g/dl)	3.3 (2.8–3.6)	3.5 (3.1–3.7)	3.2 (3.0–3.6)	3.4 (3.1–3.8)	0.43
D/P creat	0.72 (0.65–0.84)	0.72 (0.61–0.84)	0.72 (0.66–0.82)	0.70 (0.59–0.82)	0.33
BMI (kg/m^2^)	21.4 (18.8–23.3)	22.0 (19.9–24.2)	21.6 (19.7–24.1)	22.8 (20.9–25.2)	0.13
RAS inhibitors use	70	84	84	81	0.46
Icodextrin use	44	40	29	28	0.048
High glucose fluid use	7	11	11	13	0.44
Diuretics use	78	81	76	66	0.047
Dosage of PD fluid (L/day)	6.0 (4.5–6.0)	4.5 (4.5–6.0)	4.5 (4.5–6.0)	4.5 (4.5–6.0)	0.056
Peritonitis rate (times/patient-year)	0.19 (0.00–1.05)	0.00 (0.00–0.45)	0.00 (0.00–0.36)	0.00 (0.00–0.34)	0.03

Values are shown as the median (interquartile range, IQR) or a percentage. BMI: Body mass index; CVD: Cardiovascular disease; D/P creat: Dialysate-to-plasma creatinine ratio at 4 hr; eGFR: Estimated glomerular filtration rate; G1: Grade 1 hypertension; G2–3: Grade 2 & 3 hypertension; N: Normal & High normal; O: Optimal; PD: Peritoneal dialysis; RAS: Renin-angiotensin system.

**Fig 1:**
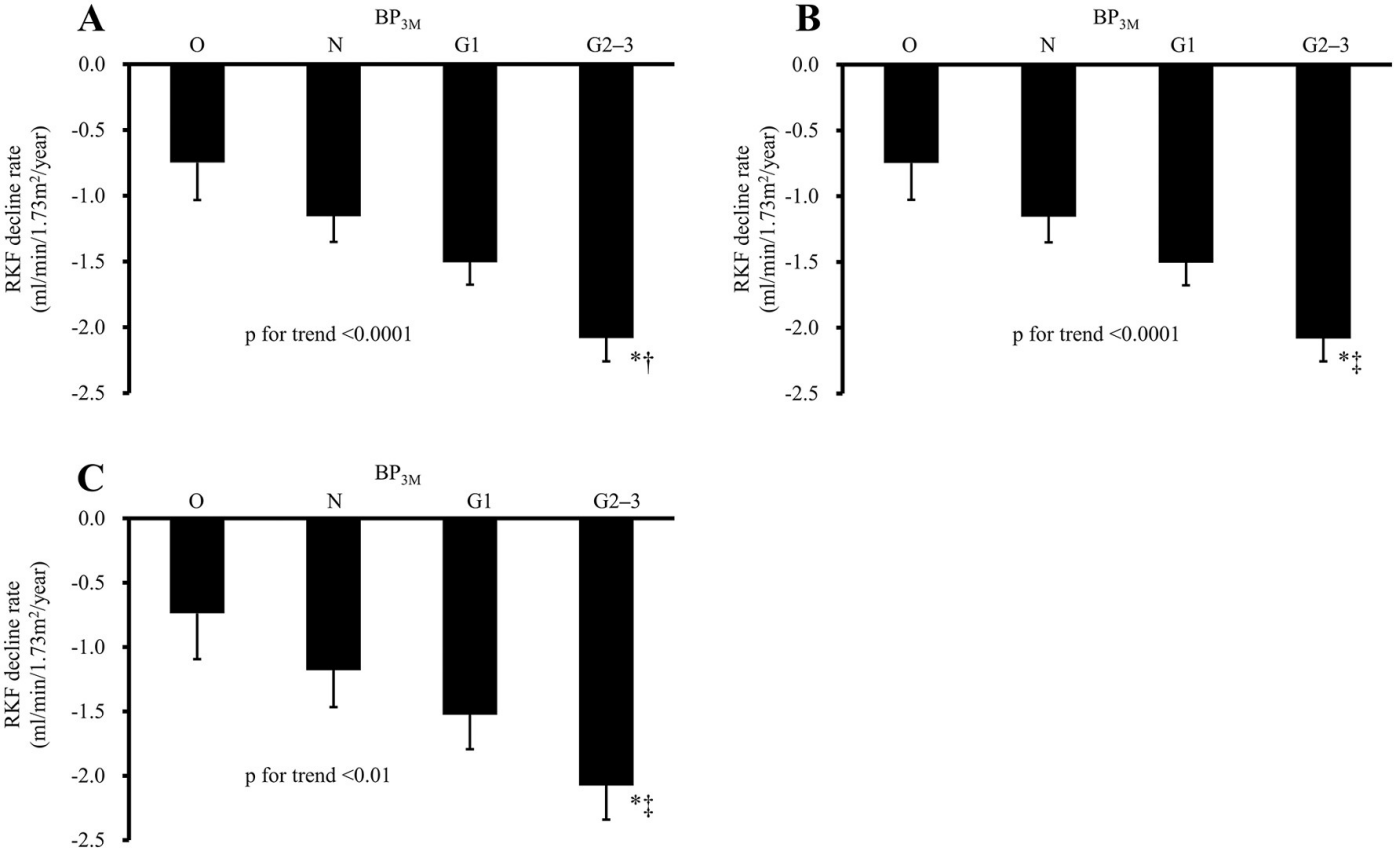
The decline rate of RKF according to the graded BP_3M_ groups. **A**: The unadjusted decline rate of RKF among the BP_3M_ groups. **B**: The sex- and age-adjusted decline rate of RKF among the BP_3M_ groups. **C**: The multivariable-adjusted decline rate of RKF among the BP_3M_ groups. *p<0.01 vs. O, †p<0.01 vs. N, ‡p <0.05 vs. N. Adjusted for age, sex, diabetes mellitus, previous history of cardiovascular disease, eGFR, hemoglobin, serum albumin, dialysate-to-plasma creatinine ratio at 4 hr, BMI, renin-angiotensin system inhibitors use, icodextrin use, high glucose fluid use, diuretics use, dosage of PD fluid, and peritonitis rate. Error bars indicate the standard error. BP_3M_: Blood pressure after 3 months of PD initiation; G1: Grade 1 hypertension; G2–3: Grade 2 & 3 hypertension; N: Normal & High normal; O: Optimal; RKF: Residual kidney function.

The differences in the decline rates of RKF and log RKF among the BP_3M_ levels were analyzed. The unadjusted and the sex- and age-adjusted decline rates of RKF gradually decreased with higher BP_3M_ levels (p for trend <0.0001; Fig 1A and 1B). This association remained unchanged after adjusting for the following factors: diabetes, previous history of CVD, eGFR, hemoglobin, albumin, D/P creat, BMI, renin-angiotensin system inhibitors use, icodextrin use, high-glucose fluid use, diuretics use, dosage of PD fluid and peritonitis rate (Fig 1C). The multivariable-adjusted rate of the decline in RKF was significantly lower in the G2–3 group compared to the O group, and in the G2–3 group compared to the N group (both p<0.05).

**Fig 2:**
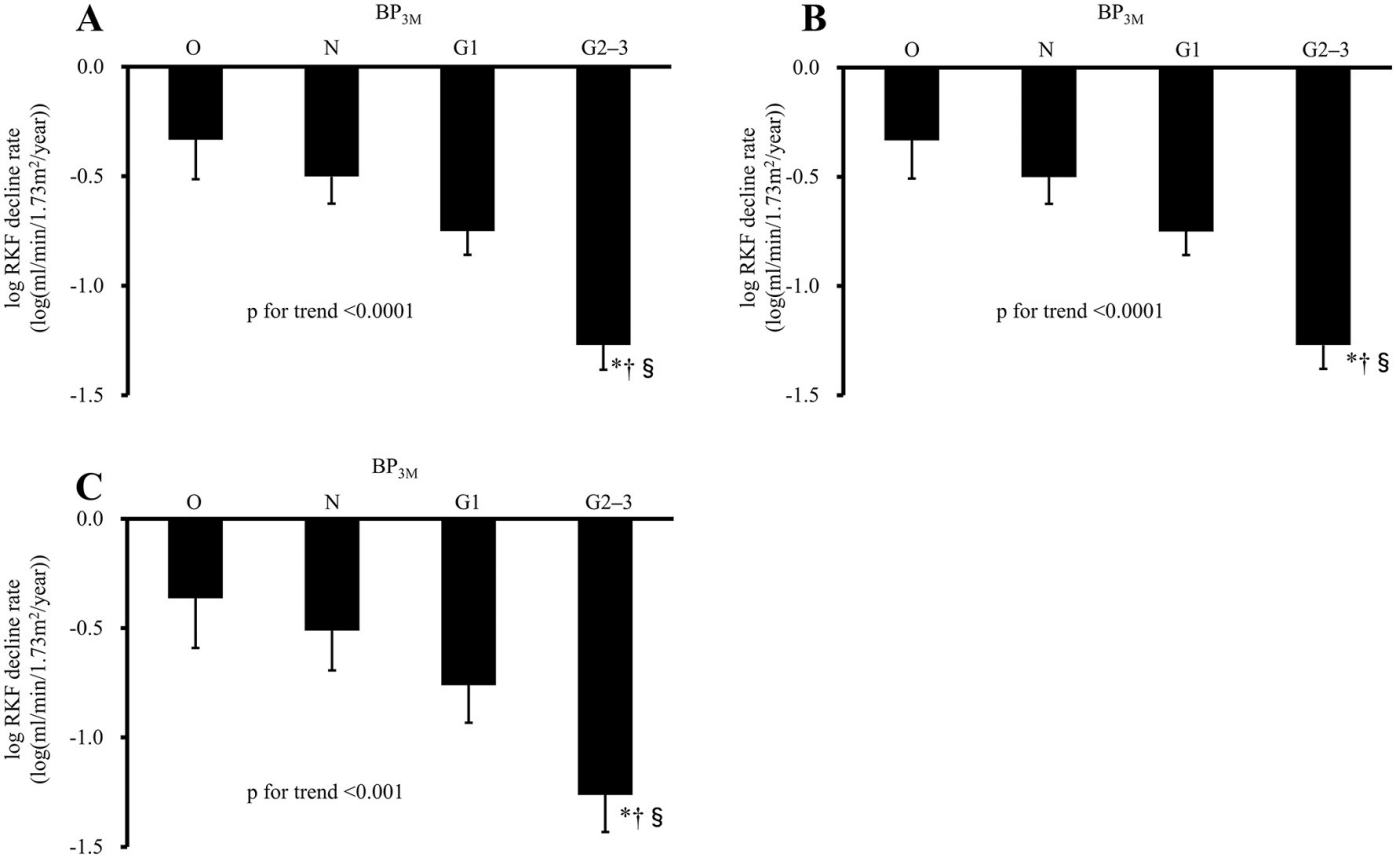
The decline rate of log RKF according to the graded BP_3M_ groups. **A**: The unadjusted decline rate of log RKF among the BP_3M_ groups. **B**: The sex- and age-adjusted decline rate of log RKF among the BP_3M_ groups. **C**: The multivariable-adjusted decline rate of log RKF among the BP_3M_ groups. *p<0.01 vs. O, †p<0.01 vs. N, §p <0.05 vs. G1. Adjusted covariates are as in Fig 1. Error bars indicate the standard error. Abbreviations are explained in the Fig 1 legend.

The unadjusted, sex- and age-adjusted, and multivariable-adjusted decline rate of log RKF also decreased with higher BP_3M_ levels (p for trend <0.001; Fig 2A–2C, respectively). The multivariable-adjusted decline rate in log RKF was significantly lower in the G2–3 group compared to the O group, and in the G2–3 group compared to the N group (all p<0.05).

We subjected the patients’ BP levels just before PD initiation (preBP) to the same analysis as the BP_3M_ levels (S1 and S2 Figs). There were 27, 57, 68 and 76 subjects in the O, N, G1 and G2–3 groups, respectively. An association between preBP and the decline rates of RKF was observed in the sex- and age-adjusted analysis (S1B Fig), but the association disappeared in the multivariable-adjusted analysis. There was no significant association between the preBP and the decline rate of log RKF. We thus considered that preBP was not as strongly correlated with RKF as BP_3M_.

A J-shaped association between BP and mortality has been observed in the PD population. We thus analyzed another group of patients, i.e., those with the lowest BP_3M_. Among the patients in the original Optimal group, patients with systolic blood pressure < 100 mmHg were regrouped as Optimal 1 (O1) and the others were classified as Optimal 2 (O2). We also analyzed the five groups of O1, O2, N, G1, and G2-3. Only five patients were included in O1 group; the remaining 22 patients were in the O2 group. The mean RKF decline rate was lower in the O1 group compared to the O2 group, but this association disappeared in the log RKF decline rate. There were no significant differences between the O1 and O2 groups in RKF or log RKF decline rate (S3 and S4 Figs).

We assessed the OR for a faster decline in RKF, which we defined as the faster side separated by the median decline rate of RKF (Table 2). The incidence of a faster decline in RKF in each of the BP groups increased with higher BP_3M_ levels, to 29.6% in O, 38.6% in N, 54.7% in G1, and 62.3% in G2–3 patients. The sex- and age-adjusted and multivariable-adjusted ORs gradually and significantly increased with higher BP_3M_ levels (p for trend <0.05). The multivariable-adjusted OR for a faster decline in RKF was 4.04 (95% CI: 1.24–13.2) in the G2–3 group compared to the O group.

**Table 2:** Multivariable-adjusted ORs for faster decline rates of RKF and log RKF.

			Unadjusted			Age- and sex- adjusted			Multivariable-adjusted^a^		
BP_3M_ levels (mmHg)	No. events	No. subjects	OR (95% CI)	p	P for trend	OR (95% CI)	p	P for trend	OR (95% CI)	p	P for trend
**RKF faster decline**^b^											
**O**	8	27	Reference		0.0059	Reference		0.0051	Reference		0.037
**N**	22	57	1.49 (0.56–3.99)	0.424		1.60 (0.60–4.52)	0.348		1.87 (0.58–6.01)	0.296	
**G1**	41	75	2.86 (1.12–7.35)	0.029		2.96 (1.18–7)	0.021		3.49 (1.14–10.7)	0.028	
**G2**–**3**	43	69	3.93 (1.51–10.2)	0.005		4.23 (1.65–11.7)	0.002		4.04 (1.24–13.2)	0.021	
**log RKF faster decline**^b^											
**O**	7	27	Reference		<0.001	Reference		<0.001	Reference		0.0042
**N**	20	57	1.54 (0.56–4.28)	0.403		2.14 (0.71–6.40)	0.551		1.84 (0.54–6.29)	0.328	
**G1**	43	75	3.83 (1.45–10.2)	0.007		5.07 (1.77–14.5)	0.003		4.61 (1.42–14.9)	0.011	
**G2**–**3**	44	69	5.03 (1.87–13.5)	0.001		6.85 (2.36–19.9)	<0.001		5.50 (1.58–19.2)	0.007	

^a^Adjusted for age, sex, diabetes mellitus, estimated glomerular filtration rate, serum albumin, dialysate-to-plasma creatinine rate at 4 hr, renin-angiotensin system inhibitors use, icodextrin use, high glucose fluid use, and peritonitis rate.

^b^Faster decline was defined as the faster side separated by the median decline rate of RKF and log RKF.

BP_3M_: Systolic blood pressure after 3 months of peritoneal dialysis initiation; CI: Confidence interval; G1: Grade 1 hypertension; G2-3: Grade 2 & 3 hypertension; N: Normal & High normal; O: Optimal; OR: Odds ratio.

We performed the same analysis for a faster decline in log RKF. The incidence of a faster decline in log RKF was 25.9% for O, 35.1% for N, 57.3% for G1, and 63.8% for G2–3 patients, with a linear trend (p for trend <0.001). This association remained unchanged after adjusting for the confounding factors. The multivariable-adjusted OR for a faster decline in log RKF was 5.50 (1.58–19.2) in the G2–3 group compared to the O group.

We assessed the multivariable-adjusted mean decline rate of RKF and that of log RKF with the 95% CI in the subgroups stratified by sex, age, and presence/absence of diabetes (Tables 3 and 4). There were no significant differences among the subgroups in the association between the decline rate of RKF and BP_3M_ levels, or between the decline rate of log RKF and BP_3M_ levels (p for interaction >0.298 for all).

**Table 3:** Multivariable-adjusted mean decline rate of RKF with 95%CI in the subgroups of BP_3M_ category.

	O	N	G1	G2–3	P for trend	P for interaction
**Sex**						0.837
Men (n = 147)	-0.843 (-1.241, 0.817)*	-1.264 (-1.695, -0.242)	-1.712 (-1.776, -0.405)	-2.310 (-2.532, -1.090)	0.023	
Women (n = 81)	-0.538 (-2.035, 0.336)	-0.987 (-2.323, -0.375)	-1.054 (-2.480, -0.470)	-1.818 (-2.963, -1.125)	0.086	
**Age**						0.305
≥ 65 years (n = 101)	-0.745 (-1.051, 0.610)*	-1.222 (-1.344, -0.199)*	-0.869 (-1.097, -0.027)*	-1.762 (-2.040, -0.936)	0.0053	
< 65 years (n = 127)	-0.732 (-1.856, 0.472)	-1.131 (-1.768, 0.364)*	-1.973 (-2.535, -0.449)	-2.299 (-2.978, -0.932)	0.0470	
**Diabetes**						0.408
No (n = 150)	-0.302 (-0.794, 1.226)*	-1.151 (-1.226, 0.192)*	-1.262 (-1.396, -0.014)*	-2.089 (-2.175, -0.830)	0.001	
Yes (n = 78)	-1.245 (-3.360, -0.525)	-1.265 (-2.694, -0.326)	-1.934 (-3.197, -1.104)	-2.050 (-3.226, -0.913)	0.671	

*p<0.05 vs. G2-3. Adjusted for age, sex, diabetes mellitus, previous history of cardiovascular disease, eGFR, hemoglobin, serum albumin, dialysate-to-plasma creatinine rate at 4 hr, BMI, renin-angiotensin system inhibitors use, icodextrin use, high glucose fluid use, diuretics use, dosage of peritoneal dialysis fluid, and peritonitis rate. CI: Confidence interval; G1: Grade 1 hypertension; G2–3: Grade 2 & 3 hypertension; N: Normal & High normal; O: Optimal.

**Table 4:** Multivariable-adjusted mean decline rate of log RKF with 95%CI in the subgroups of BP_3M_ category.

	O	N	G1	G2–3	P for trend	P for interaction
**Sex**						0.891
Men (n = 147)	-0.434 (-0.933, 0.296)*	-0.529 (-1.003, -0.136)*	-0.817 (-0.999, -0.180)*	-1.231 (-1.558, -0.698)	0.028	
Women (n = 81)	-0.230 (-1.228, 0.563)*	-0.470 (-1.642, -0.171)	-0.620 (-1.810, -0.292)	-1.298 (-2.177, -0.789)	0.024	
**Age**						0.398
≥ 65 years (n = 101)	-0.320 (-0.745, 0.314)	-0.399 (-0.564, 0.167)*	-0.397 (-0.654, 0.030)	-0.736 (-1.076, -0.371)	0.030	
< 65 years (n = 127)	-0.396 (-1.232, 0.263)*	-0.640 (-1.243, 0.126)*	-1.009 (-1.564, -0.224)*	-1.6s37 (-2.214, -0.900)	0.007	
**Diabetes**						0.298
No (n = 150)	-0.248 (-0.719, 0.575)*	-0.442 (-0.843, 0.140)*	-0.572 (-0.932, 0.026)*	-1.312 (-1.624, -0.684)	0.0005	
Yes (n = 78)	-0.499 (-1.898, -0.431)	-0.719 (-1.502, -0.276)	-1.054 (-1.787, -0.704)	-1.162 (-1.818, -0.621)	0.608	

*p<0.05 vs. G2-3. Adjusted for age, sex, diabetes mellitus, previous history of cardiovascular disease, eGFR, hemoglobin, serum albumin, dialysate-to-plasma creatinine rate at 4 hr, BMI, renin-angiotensin system inhibitors use, icodextrin use, high glucose fluid use, diuretics use, dosage of peritoneal dialysis fluid, and peritonitis rate. CI: Confidence interval; G1: Grade 1 hypertension; G2–3: Grade 2 & 3 hypertension; N: Normal & High normal; O: Optimal.

The VIF of all parameters was <2.1, which indicated that there was no multicollinearity.

## Discussion

Our analyses revealed that the decline rate of RKF was negatively correlated with BP_3M_ levels. This association remained unchanged even after adjustment for the confounding factors. In the multivariable-adjusted logistic regression analysis, the OR for a faster decline of RKF gradually increased with higher BP_3M_ levels. The same relationship was observed between the OR for a faster decline of log RKF and higher BP_3M_ levels. This indicates that the higher the BP_3M_ level is, the faster the decline of RKF is. We therefore speculate that optimal BP control is important for the preservation of kidney function not only before dialysis therapy but also after PD initiation. Our results indicate that the 2018 ESC/ESH classification of office BP and definitions of hypertension grade can be applied to PD patients in terms of RKF preservation.

Hypertension is a strong risk factor for the onset and deterioration of CKD [14], and thus treatment of hypertension is the basis of the control of CKD progression. Anti-hypertensive therapy inhibits the progression of CKD [15–17] and has been shown to reduce both the risk of CVD onset and the risk of CVD-related mortality [25]. However, all of these findings were obtained from patients who had not yet started PD therapy; it thus remains to be determined whether these findings are also applicable for patients who are undergoing PD. There have been few reports with respect to the association between the decline of RKF and BP levels in patients who are undergoing PD. Menon et al. reported that BP control deteriorated steadily with time and was associated with the decline of RKF [26]. In their study using a logistic regression analysis, Johnson et al. reported that a higher BP level was not associated with a faster decline in RKF [27]. To the best of our knowledge, the present study is the first to report a significant association between BP levels and the decline rate of RKF in PD patients.

We used the parameter of blood pressure after 3 months of PD initiation as a predictor of RKF decline, and our findings revealed that this parameter was significantly associated with the decline rate of RKF. However, blood pressure just before PD initiation was not associated with the decline rate of RKF, which was also reported in several previous investigations [12, 27, 28]. Other studies have noted that PD patients displayed blood pressure variations related to dynamic changes in their fluid volume status for the first 2 months after the start of PD [29, 30]. We suspect that those patients’ blood pressure changed greatly in 3 months, and that after 3 months of PD initiation these changes impacted the RKF decline.

The International Society for Peritoneal Dialysis (ISPD) guidelines indicate that control of volume overload is the first line of hypertension treatment in PD patients [31]. Therefore, measuring the blood pressure of PD patients after their fluid volume status has stabilized, rather than at the time of PD initiation, might be useful for predicting an RKF decline. Previous multivariable study included BP at PD initiation as a variable but did not include BP at the stable fluid volume status after PD initiation [27]. We thus suspect that the decline in RKF was predicted not by BP levels at the time of PD initiation, but by BP levels after their fluid volume status had stabilized.

We calculated the decline rate of the logarithmic scale of RKF and analyzed it in the same way as the decline rate of RKF. A faster decline rate of log RKF remained associated with higher BP_3M_ levels, as did the decline rate of RKF. Several studies have reported that the decline rate of RFK is exponential rather than linear [12, 23, 24]. If the rate of RKF decline is calculated using a linear regression although the RKF actually declines exponentially, the rate of RKF decline among patients with higher baseline eGFR values will be overestimated. Some studies that calculated the RKF decline rate by using a linear regression showed that a higher baseline eGFR was a risk factor for an increased rate of RKF decline [19, 27, 31]. To solve this problem, we used the decline rate of log RKF. We observed that the BP_3M_ levels remained associated with not only the decline rate of RKF but also that of log RKF. There is a possibility that the decline rate of log RKF represents the decline of RKF more accurately in PD patients. In other cohort studies, the decline of RKF was assessed based on kidney survival before the patient’s urine volume decreased to a specific volume or less (for example, <100 mL). Therefore, the interval until an endpoint is reached can be shortened in patients with delayed PD initiation. If a decrease in urine volume is established as an outcome of a kidney survival analysis, a low eGFR might become a risk factor. We therefore did not use the urine volume as an outcome.

Many studies have consistently shown a J-shaped relationship between mortality and lowest and highest BP in CKD G5D patients [32]. This relationship also applies to PD patients, without exception [33]. Our additional analysis revealed no association between the lowest BP (O1) and O2 groups. Although the average RKF decline rate was lower in the O1 group than the O2 group, this association disappeared in the log RKF decline rate. The small number of patients in the O1 group (n = 5) may be related to the lack of significant differences. If a relationship between low blood pressure and a faster decline rate of RKF is established, it will be very helpful in understanding the poor prognoses in patients with the lowest BP among patients undergoing PD. Further larger-scale studies are needed to investigate this relationship.

This study has several limitations. First, it was a retrospective observational study with a small number of samples, and the data were obtained from two hospitals. Confounding factors might also have influenced the results. It might not be possible to apply our findings to PD patients from other hospitals. In the future, the decline rate of RKF should be evaluated in a large, multicenter, prospective study. Second, there might have been inter-individual differences in the accuracy of the RKF slopes, because neither the follow-up period nor the frequency of RKF measurement was standardized among the patients when we calculated the RKF slope. These two parameters should be standardized among subjects to produce accurate RKF slope data. In addition, almost all of the samples examined herein were brought to the outpatient clinic. Although it is probable that most patients were able to store urine correctly based on the detailed explanation of urine collection that were given, it cannot be denied that urine may not have been stored accurately in some samples and patients. Third, the patients’ BP_3M_ levels were measured only once during the outpatient visit. Since these levels were not measured multiple times, they may not reflect the accurate BP levels. Fourth, the parameters used in multivariable-adjusted analysis did not include parameters related to fluid volume, such as the cardiothoracic ratio or echocardiography findings.

In conclusion, our study revealed for the first time that a higher blood pressure level, not just before PD initiation but after 3 months of PD initiation was associated with a slower RKF decline. Appropriate blood pressure control might be important for maintaining RKF not only before dialysis therapy but also after PD initiation.

## Supporting information

S1 Fig The decline rate of RKF according to the graded preBP groups. **A**: The unadjusted decline rate of RKF among the preBP groups. **B**: The sex- and age-adjusted decline rate of RKF among the preBP groups. **C**: The multivariable-adjusted decline rate of RKF among the preBP groups. *p<0.01 vs. O. Adjusted covariates are as in Fig 1. Error bars indicate the standard error. n.s.: Not significant; preBP: Blood pressure levels just before PD initiation. Other abbreviations are explained as in Fig 1 legend.
(TIF)

S2 Fig The decline rate of log RKF according to the graded preBP groups. **A**: The unadjusted decline rate of log RKF among the preBP groups. **B**: The sex- and age-adjusted decline rate of log RKF among the preBP groups. **C**: The multivariable-adjusted decline rate of log RKF among the preBP groups. Adjusted covariates are as in Fig 1. Error bars indicate the standard error. n.s.: Not significant; preBP: Blood pressure levels just before PD initiation. Other abbreviations are explained as in Fig 1 legend.
(TIF)

S3 Fig The decline rate of RKF according to the graded BP_3M_ groups. **A**: The unadjusted decline rate of RKF among the BP_3M_ groups. **B**: The sex- and age-adjusted decline rate of RKF among the BP_3M_ groups. **C**: The multivariable-adjusted decline rate of RKF among the BP_3M_ groups. *p<0.01 vs. O2, †p<0.05 vs. O2. Adjusted covariates are as in Fig 1. Error bars indicate the standard error. O1: Optimal 1; O2: Optimal 2. Other abbreviations are explained as in Fig 1 legend.
(TIF)

S4 Fig The decline rate of log RKF according to the graded BP_3M_ groups. **A**: The unadjusted decline rate of log RKF among the BP_3M_ groups. **B**: The sex- and age-adjusted decline rate of log RKF among the BP_3M_ groups. **C**: The multivariable-adjusted decline rate of log RKF among the BP_3M_ groups. *p<0.01 vs. O2. Adjusted covariates are as in Fig 1. Error bars indicate the standard error. O1: Optimal 1; O2: Optimal 2. Other abbreviations are explained as in Fig 1 legend.
(TIF)
