# Supplementary figures and images for: Association of blood pressure after peritoneal dialysis initiation with the decline rate of residual kidney function in newly-initiated peritoneal dialysis patients
Source: PLoS One. 2021 Jul 8;16(7):e0254169. doi: 10.1371/journal.pone.0254169 (PMC8266121; doi:10.1371/journal.pone.0254169)

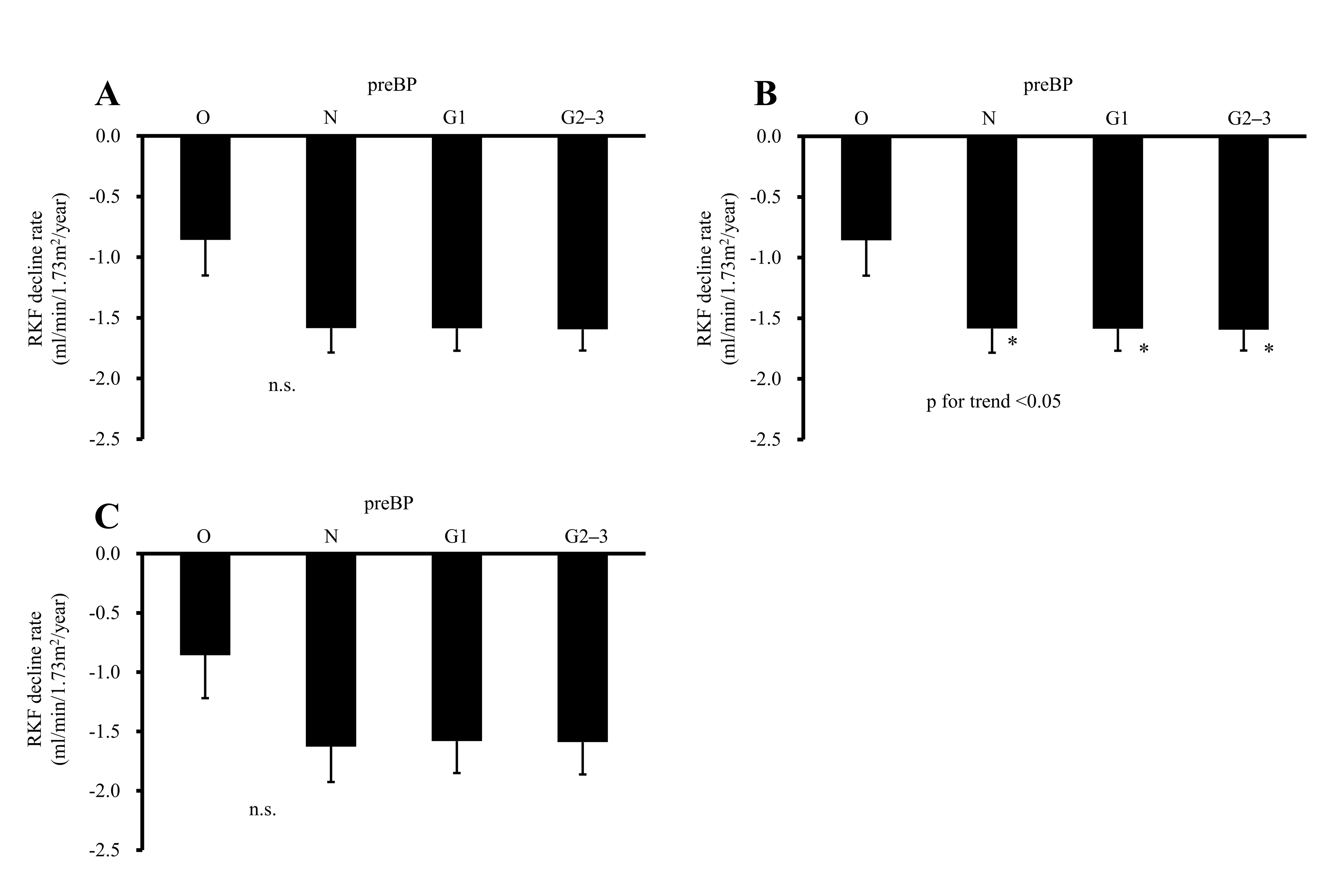

Supplement: S1 Fig — A: The unadjusted decline rate of RKF among the preBP groups. B: The sex- and age-adjusted decline rate of RKF among the preBP groups. C: The multivariable-adjusted decline rate of RKF among the preBP groups. *p<0.01 vs. O. Adjusted covariates are as in Fig 1. Error bars indicate the standard error. n.s.: Not significant; preBP: Blood pressure levels just before PD initiation. Other abbreviations are explained as in Fig 1 legend. (TIF) [file pone.0254169.s001.tif]

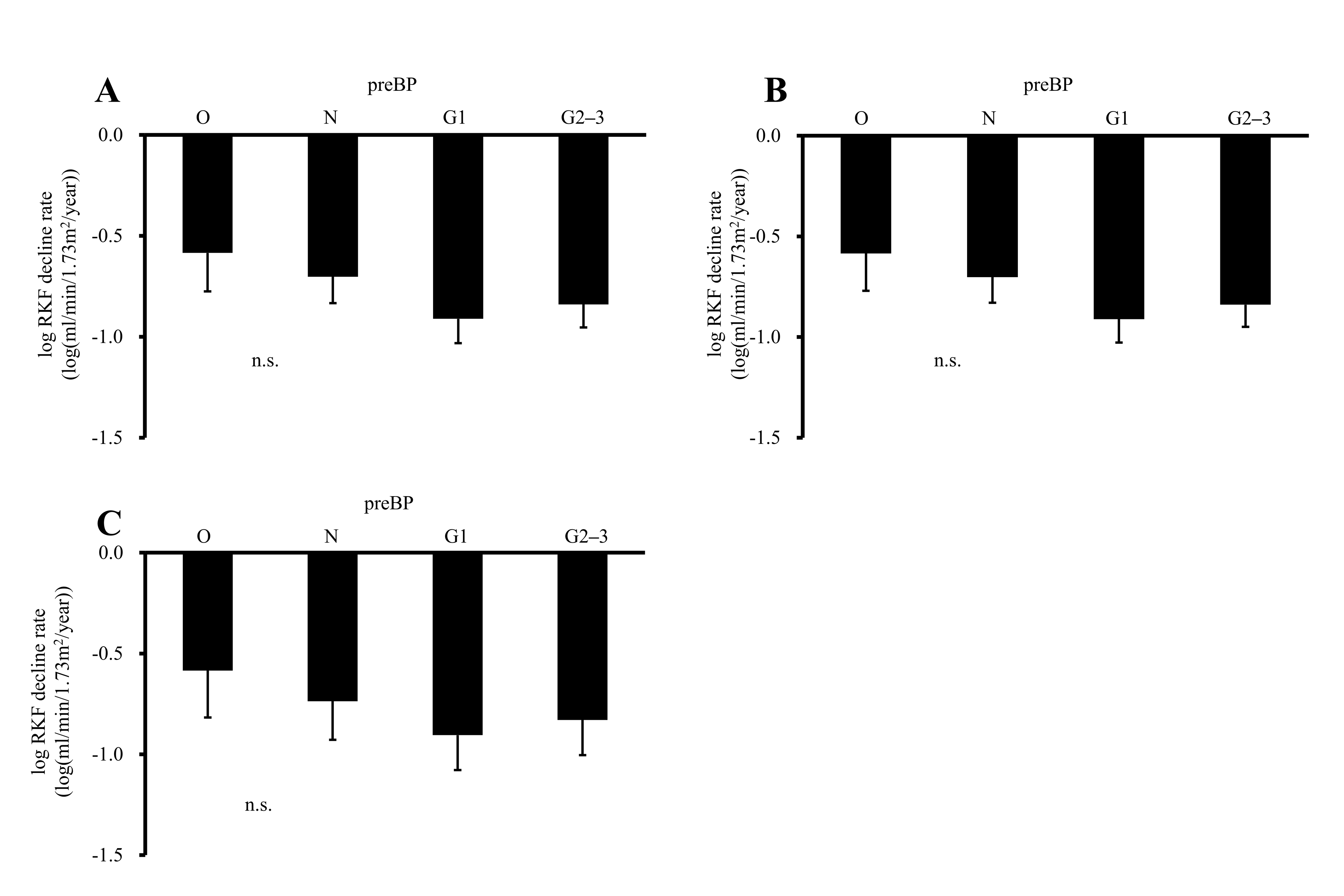

Supplement: S2 Fig — A: The unadjusted decline rate of log RKF among the preBP groups. B: The sex- and age-adjusted decline rate of log RKF among the preBP groups. C: The multivariable-adjusted decline rate of log RKF among the preBP groups. Adjusted covariates are as in Fig 1. Error bars indicate the standard error. n.s.: Not significant; preBP: Blood pressure levels just before PD initiation. Other abbreviations are explained as in Fig 1 legend. (TIF) [file pone.0254169.s002.tif]

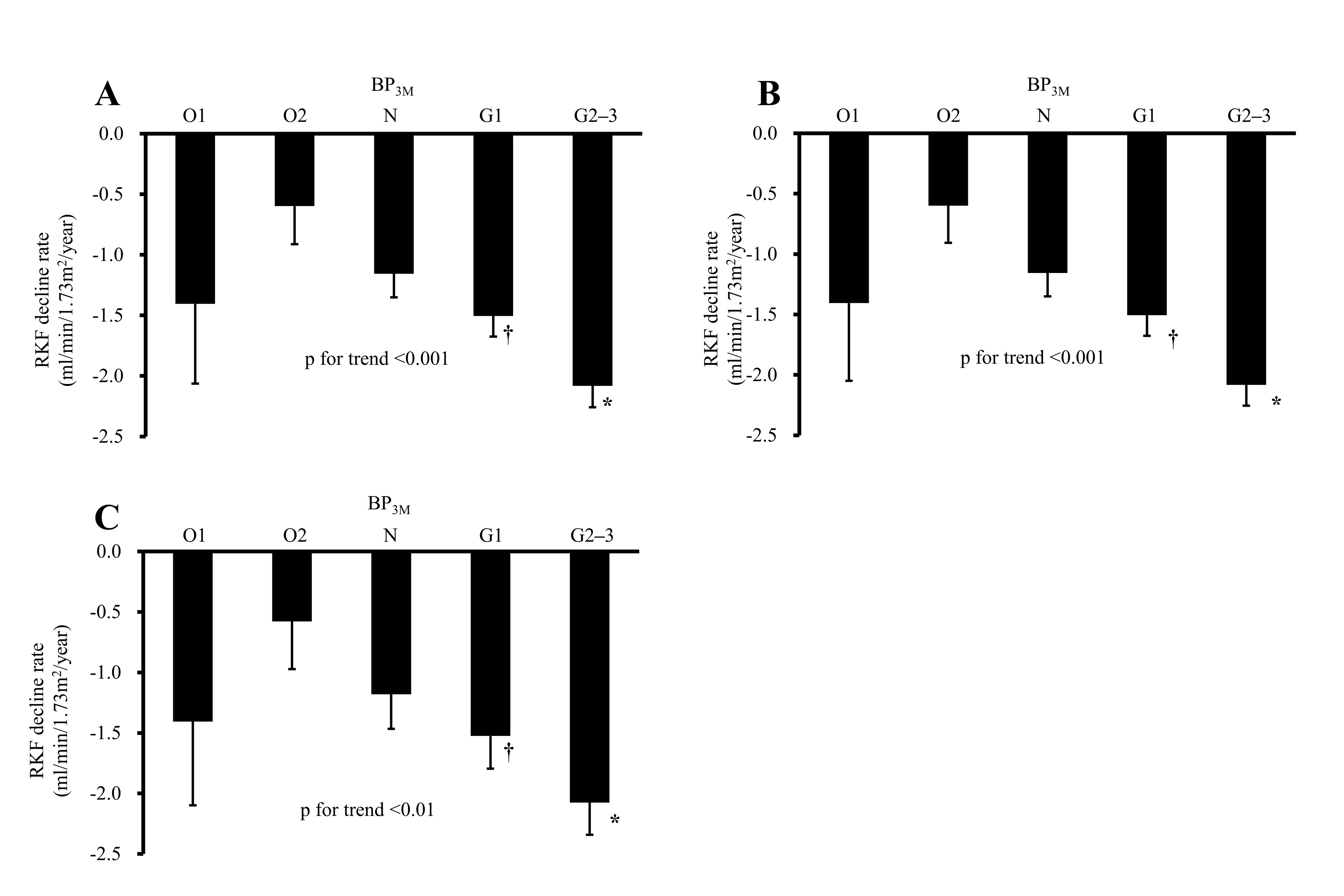

Supplement: S3 Fig — A: The unadjusted decline rate of RKF among the BP3M groups. B: The sex- and age-adjusted decline rate of RKF among the BP3M groups. C: The multivariable-adjusted decline rate of RKF among the BP3M groups. *p<0.01 vs. O2, †p<0.05 vs. O2. Adjusted covariates are as in Fig 1. Error bars indicate the standard error. O1: Optimal 1; O2: Optimal 2. Other abbreviations are explained as in Fig 1 legend. (TIF) [file pone.0254169.s003.tif]

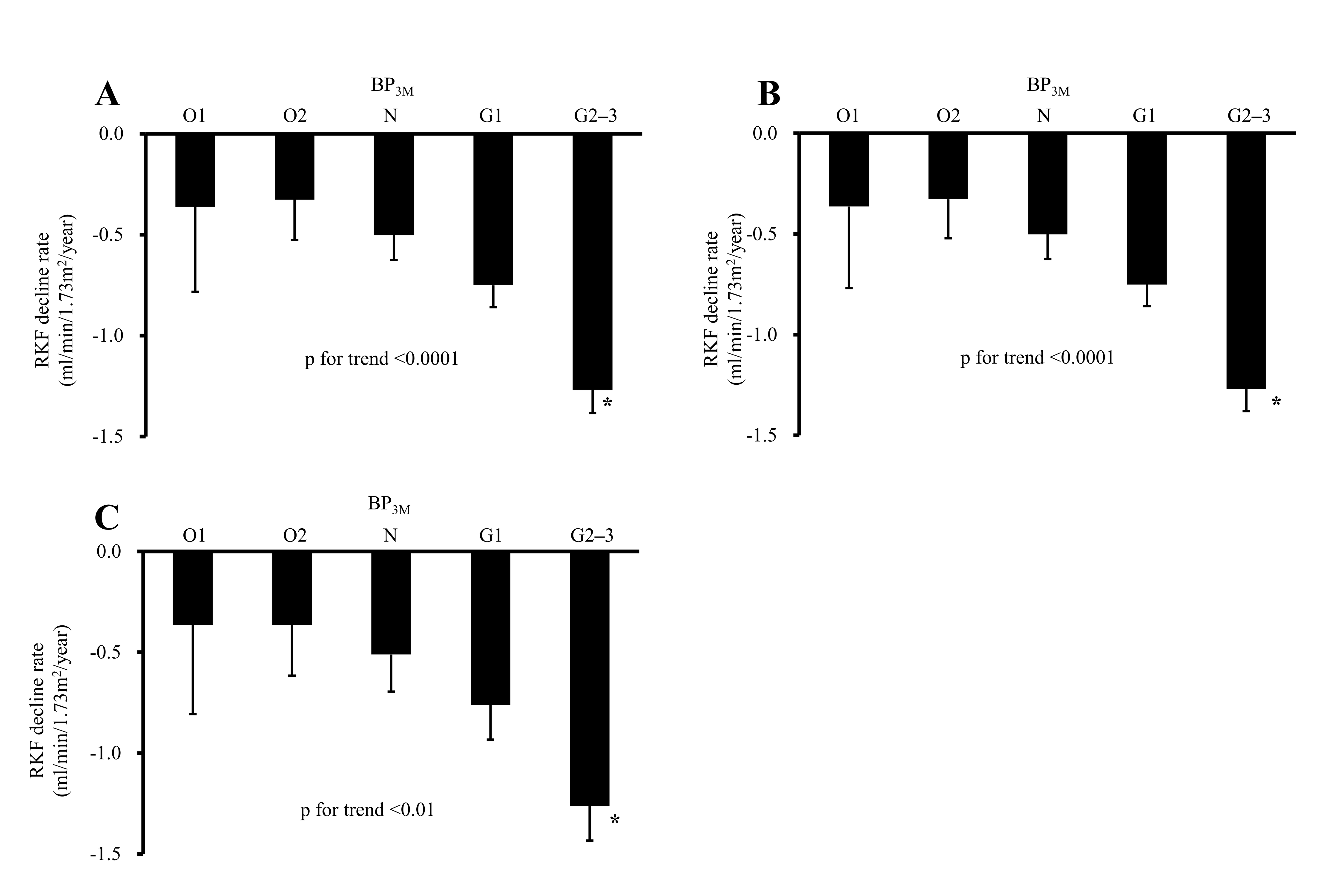

Supplement: S4 Fig — A: The unadjusted decline rate of log RKF among the BP3M groups. B: The sex- and age-adjusted decline rate of log RKF among the BP3M groups. C: The multivariable-adjusted decline rate of log RKF among the BP3M groups. *p<0.01 vs. O2. Adjusted covariates are as in Fig 1. Error bars indicate the standard error. O1: Optimal 1; O2: Optimal 2. Other abbreviations are explained as in Fig 1 legend. (TIF) [file pone.0254169.s004.tif]
